# Employee perceptions and experiences with tirzepatide treatment for obesity or overweight in the US: Insights from the PERCEPTIONS Survey

**DOI:** 10.1016/j.obpill.2026.100298

**Published:** 2026-07-09

**Authors:** Theresa Hunter Gibble, Tamara Al-Zubeidi, Claire Gerber, Elizabeth Collins, Aikaterini Vardavoulia, Alexandra Lin, Xuanyao He, Michael Shepherd, Angela Fitch, Harold Bays

**Affiliations:** a Eli Lilly and Company, Indianapolis, IN, USA; b Clarivate, London, UK; c Knownwell, Needham, MA, USA; d Monroe Biomedical Research, Louisville Metabolic and Atherosclerosis Research Center, Louisville, KY, USA

**Keywords:** Full-time employees, Obesity, Overweight, Tirzepatide, Insurance, Employer

## Abstract

**Objective:**

To characterize real-world experiences, perceptions, and workplace-related outcomes among full-time employed adults in the United States with obesity or overweight initiating tirzepatide for obesity management, and to assess perceptions of employer-provided insurance coverage for obesity medications (OMs).

**Methods:**

This analysis reports baseline findings from the PERCEPTIONS survey, an observational, longitudinal, real-world study conducted in the United States. Full-time employed adults (≥18 years) with obesity (body mass index [BMI] ≥30 kg/m^2^) or overweight (BMI ≥27 kg/m^2^ with ≥1 obesity related complication [ORC]) initiating tirzepatide for obesity management were recruited via clinical sites and specialist recruitment agencies and completed a self-administered electronic survey (June to November 2025). Demographic, clinical, and employment characteristics; engagement in employer-sponsored wellness programs; perceptions of employer-provided insurance coverage for OMs; and patient-reported outcomes, including work productivity and activity impairment (WPAI) assessed using the WPAI questionnaire were reported.

**Results:**

Among the 518 full-time employees included, mean (SD) age was 46.0 (11.6) years, BMI was 38.4 (8.4) kg/m^2^, and mean employment duration was 89.8 months (∼7.5 years). Most participants had employer-provided health insurance (80.9%), among these, 55.9% reported coverage for OMs. Majority (96.5%) believed employer-provided insurance should include OM coverage, citing improved health, productivity, and management of obesity-related complications. 81.7% reported that OM coverage may increase job satisfaction and 52.3% reported that they would consider changing jobs in order to receive OM coverage. WPAI results indicated substantial impairment, with mean activity impairment of 43.0% and overall work impairment of 32.4%, driven primarily by presenteeism.

**Conclusion:**

Among full-time employees initiating tirzepatide, obesity was associated with considerable work and activity impairment, and strong support was expressed for employer-provided OM coverage. These findings highlight the potential clinical and functional value of obesity pharmacotherapy and may inform employer and insurer policies aimed at reducing barriers to care and supporting long-term obesity management.

## Graphical abstract

**Figure:**
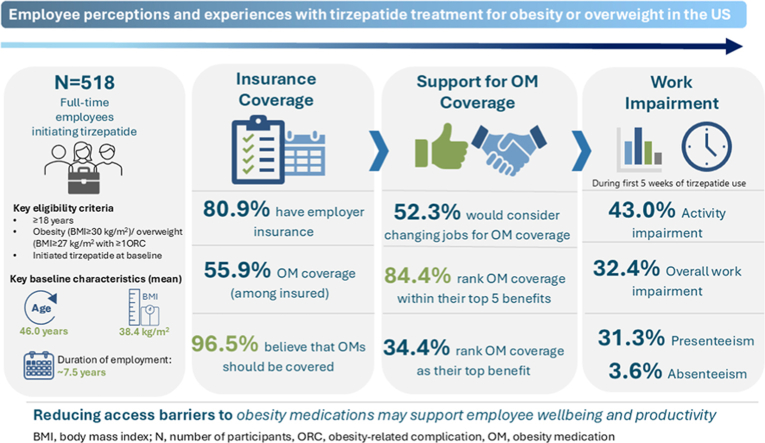

## 1 Introduction

The obesity epidemic affects nearly 3 billion adults globally and contributes to significant health, economic, and psychosocial burdens [1,2]. Despite the availability of evidence-based therapies (e.g., obesity medications [OMs], bariatric surgery, healthful nutrition, routine physical activity, and behavior modification), obesity management remains suboptimal in people with obesity [3,4]. Recently, OMs, such as tirzepatide, a once-weekly glucose-dependent insulinotropic polypeptide (GIP) and glucagon-like peptide-1 receptor agonist (GLP-1 RA), have demonstrated efficacy in reducing weight and improving cardiometabolic risk factors in multiple phase 3 clinical trials and real-world studies, leading to a growing interest in the real-world use of tirzepatide [[5], [6], [7], [8], [9], [10], [11]].

While clinical trials provide robust data on the safety and efficacy of treatment, understanding individuals’ perceptions and experiences in real-world settings is important for optimizing treatment adherence and health outcomes. Full-time employees with obesity represent a unique population where obesity management intersects with workplace productivity, health benefits, and quality of life [12]. Previous studies have found that obesity negatively impacts workplace productivity, with outcomes varying depending on the type of occupation, primarily due to its considerable clinical burden and related socioeconomic challenges [13]. Specifically, employees with obesity in the United States (US) reported significantly greater absenteeism compared to those with a normal body mass index (BMI), in addition to health-related work impairment, disability pension, and early retirement [14,15]. Further, excess weight was often associated with work performed at reduced capacity, also referred to as presenteeism or lost on-the-job productivity [14,15]. These trends highlight the substantial burden of obesity not only as a medical condition but also as a significant factor influencing workplace functioning and economic outcomes. Thus, it may be beneficial for employers to offer coverage for OMs, which may decrease direct and indirect costs associated with obesity and improve employee wellbeing and productivity [16].

Additionally, assessing employees' perceptions of employer-based benefits and their engagement in employer-sponsored wellness programs may help understand employees’ behavior, adherence to treatment, and overall treatment outcomes. This is particularly relevant for employed adults who may face unique work-related challenges and insurance constraints. There is limited research that has explored how full-time employees perceive and experience treatment with tirzepatide in the real world, including factors influencing treatment adherence and satisfaction. To address this gap, this real-world study aimed to explore the experience of full-time employees initiating tirzepatide in the US.

## 2 Methods

### 2.1 Study design and study population

This study reports the findings of the baseline time point of an observational, longitudinal survey-based study (PERCEPTIONS). Data was collected from June 2025 to November 2025, using a structured, electronic initiation survey administered to eligible participants following electronic informed consent. The survey was developed with feedback from health-care providers and individuals with obesity. It examined real-world employment characteristics, employer-sponsored benefits, wellness programs, and patient-reported outcomes (PROs) among full-time US adults with obesity or overweight initiating tirzepatide for obesity management (specifically, Zepbound), using prospectively collected electronic survey data under rigorous ethical and regulatory standards.

The study included OM-eligible adults (≥18 years) living with obesity (BMI ≥30 kg/m^2^) or overweight (BMI ≥27 kg/m^2^ with at least one obesity-related complication [ORC]) initiating tirzepatide at baseline. Eligible participants were employed full-time at the time of tirzepatide initiation (within eight weeks of first administration). Priori power calculations were used to estimate a sample size, which resulted in a target of at least N = 500. People with type 2 diabetes (T2D), prior bariatric surgery, use of tirzepatide, and participation in any clinical trial for the treatment of obesity were excluded. Recruitment was conducted via clinical sites, and specialist recruitment agencies, using Western Copernicus Group Institutional Review Board-approved materials and a screening form (#20251377). The survey was administered directly to participants online. All participants provided electronic informed consent prior to any study activity.

All procedures were conducted in accordance with the Declaration of Helsinki, Good Pharmacoepidemiology Practices, and applicable US regulations. Participant confidentiality was maintained through de-identification and secure data management practices.

### 2.2 Outcomes

The survey captured demographic and clinical characteristics, as well as employment characteristics, including working hours per week, short-term disability use, annual income, employment duration, industry type, payment type, effort required at the job, and employer size. Engagement of full-time employees in workplace wellness programs and their perceptions of employer-provided insurance benefits (including treatment covered by employer, satisfaction with current job, likelihood to stay with current employer, importance of OM coverage as employee benefit, and ranking of job benefits) were described.

Additionally, the survey included both standardized PRO measures and custom questions developed by the study team, with input from an advisory committee of patients and healthcare professionals. The following validated Work Productivity and Activity Impairment (WPAI) PRO was administered at baseline:

The WPAI is a PRO measure formed of 6 items that assess the burden of disease on work productivity and regular activities. Four score types were calculated from the WPAI:

o absenteeism (work time missed)
presenteeism (impairment at work/reduced on-the-job effectiveness)
work productivity loss (overall work impairment/absenteeism plus presenteeism); and
activity impairment.

Scores were expressed as impairment percentages, with higher numbers indicating greater impairment and less productivity [17,18].

### 2.3 Statistical analysis

Descriptive statistics were calculated for quantitative data, including means, medians, standard deviations, and interquartile ranges for continuous variables, and frequencies and percentages for categorical variables. Missing data were handled according to the scoring algorithms of each validated instrument; the amount of missing data was reported for each item. No multiplicity adjustments were performed. All analyses were performed using SPSS V29.

## 3 Results

### 3.1 Demographic and clinical characteristics

A total of 518 full-time employees with obesity or overweight receiving tirzepatide were included in the analysis (Table 1). The mean (SD) age was 46.0 (11.6) years, the majority were female (78.2%) and White (69.5%), while 9.7% identified as Hispanic or Latino. The mean (SD) baseline BMI and weight were 38.4 (8.4) kg/m^2^ and 240.2 (60.1) pounds, respectively; median annual income was $68,000 (Table 1).

**Table 1:** Demographic and clinical characteristics of full-time employees initiating tirzepatide.

Characteristics	Full-time employee (N = 518)
**Age, years**	
Mean (SD)	46.0 (11.6)
**Sex, n (%)**	
Female	405 (78.2)
Male	112 (21.6)
Other	<5
**Race**^a^**, n%**	
White	360 (69.5)
Black or African American	128 (24.7)
Asian	16 (3.1)
American Indian or Alaskan Native	8 (1.5)
Native Hawaiian or other Pacific islander	<5
Other	23 (4.4)
**Ethnicity, n%**	
Hispanic or Latino	50 (9.7)
Not Hispanic or Latino	468 (90.3)
**Education, n%**	
Bachelor's degree	152 (29.3)
Graduate/Post-graduate degree	151 (29.2)
High school diploma or equivalent	117 (22.6)
Associate's degree	87 (16.8)
Other	10 (1.9)
Some high school, but no diploma	<5
**BMI, kg/m^2^**	
Mean (SD)	38.4 (8.4)
**Weight, pounds**	
Mean (SD)	240.2 (60.1)
**Description that best represents your primary full-time job, n%**	
Very heavy	6 (1.2)
Heavy work	21 (4.1)
Medium work	78 (15.1)
Light work	113 (21.8)
Sedentary work	300 (57.9)
**Working hours per week at primary full-time job**	
Mean (SD)	39.5 (8.6)
**Short-term disability from full-time job before tirzepatide initiation, n (%)**	
Yes	25 (4.8)
No	493 (95.2)
**Annual individual income (USD)**	
Mean	94,820.1 (291,314.6)
Median (IQR)	68,000.0 (45,000–96,500)
**Employment duration at current primary full-time workplace/company (months)**	
Mean (SD)	89.8 (94.5)
Median (IQR)	51.0 (26.0–121.0)
**Industry of employment, n (%)**	
Education	84 (16.2)
Hospital/Provider Systems	84 (16.2)
Professional & Business Services	38 (7.3)
Government	27 (5.2)
Retail & Wholesale Trade	27 (5.2)
Finance/Banking	21 (4.1)
Insurance	20 (3.9)
Technology	17 (3.3)
Manufacturing	16 (3.1)
Construction	14 (2.7)
Biopharma/medical device	13 (2.5)
Leisure & Hospitality	11 (2.1)
Telecommunications	9 (1.7)
Transportation, logistics, warehousing	9 (1.7)
Utilities/energy	8 (1.5)
Airlines	6 (1.2)
Defense	6 (1.2)
Aviation	<5
Information	<5
Goods-producing, excluding agriculture/utilities	<5
Media	<5
Agriculture, forestry, fishing, hunting	<5
Other	92 (17.8)
**Payment type for primary full-time work, n (%)**	
Fixed salary	253 (48.8)
Hourly rate	219 (42.3)
Commission-based	21 (4.1)
Fixed and commission-based	<5
Fixed and other	<5
Hourly and fixed	<5
Other	19 (3.7)
**Size of primary full-time employer, n (%)**	
<500	205 (39.6)
500–999	43 (8.3)
1000–4999	71 (13.7)
5000–9999	35 (6.8)
10,000–19,999	37 (7.1)
20,000–49,999	25 (4.8)
50,000–99,999	18 (3.5)
>100,000	46 (8.9)
Unsure	38 (7.3)

Values 1–4 are represented as <5.

Sedentary work: Requires the ability to sit up to 6 h in an 8-h work day, lift light objects such as files and paperwork frequently during the day, and objects weighing up to 10 pounds occasionally during the day.

Light work: Requires the ability to stand up to 6 h in an 8-h work day, lift up to 10 pounds frequently and up to 25 pounds occasionally.

Medium work: Requires the ability to stand up to 6 h in an 8-h work day, lift up to 25 pounds frequently and 50 pounds occasionally.

Heavy work: Requires the ability to stand up to 6 h in an 8-h work day, lift up to 50 pounds frequently and 100 pounds occasionally.

Very heavy work: Requires the ability to stand up to 6 h in an 8-h work day, lift >50 pounds frequently and >100 pounds occasionally. IQR, interquartile range; Min, minimum; Max, maximum.

Abbreviations: BMI, body mass index; IQR, interquartile range; N, total number of participants; n, number of participants in the specific category; SD, standard deviation; USD, United States Dollar.

a Note that participants could select more than one race, so counts exceed the sample size.

### 3.2 Employment demographics

Participants worked for an average of 39.5 h per week. Most of them (95.2%) had not taken short-term disability prior to initiating tirzepatide. The mean duration of full-time employment was 89.8 months (∼7.5 years). The most common employment industries were education (16.2%) and hospital/provider systems (16.2%). Nearly half of the participants (48.8%) were paid a fixed salary, while 42.3% earned an hourly wage. Full-time jobs were predominantly sedentary (57.9%) or involved light work (21.8%). Most commonly, the participants included in our analysis (39.6%) had less than 500 employees at their current workplace (Table 1).

### 3.3 Engagement of full-time employees with workplace wellness programs

The majority of participants (80.9%) had employer-provided health insurance, and among them, 55.9% had coverage for OMs. Approximately 82.0% of participants did not have any other non-employer healthcare insurance covering their OM. Tirzepatide was covered in 93.0% (n = 57) of participants who did not have primary employer-provided health insurance but had other insurance to cover OM (Table 2).

**Table 2:** Insurance coverage and engagement of full-time employees in workplace wellness programs.

Characteristics	Full-time employees (N = 518)
**Employer-provided health insurance from primary full-time employer**	
Yes	419 (80.9)
No	99 (19.1)
**OM covered by employer-provided health insurance**	n = 419
Yes	234 (55.9)
No	158 (37.7)
Unsure	27 (6.4)
**OM covered by any other healthcare insurance**	
Yes	76 (14.7)
No	424 (81.9)
Unsure	18 (3.5)
**Tirzepatide covered by health insurance**^a^	n = 57
Yes	53 (93.0)
No	4 (7.0)
**Wellness programs offered by primary full-time employer**	
Yes	170 (32.8)
No	235 (45.4)
Unsure	113 (21.8)
**Employees were required to enroll in primary full-time employer's wellness program to receive tirzepatide or other GLP-1 medications**^b^	n = 157
Yes	23 (14.7)
No	134 (85.4)
**Wellness program that was required to receive tirzepatide or other GLP-1 medications**^c^	n = 23
Nutritional guidance, support, or counseling	21 (91.3)
Weigh-ins or other types of health checks	17 (73.9)
Health coach	15 (65.2)
Mental health support	7 (30.4)
Telehealth	6 (26.1)
Gym membership	5 (21.7)
Specific fitness program engagement	<5
**Engaged in employer's wellness program in an attempt to lose weight**^b^	n = 170
Has engaged any time	110 (64.7)
Currently engaged	52 (30.6)
Previously engaged	58 (34.1)
Never engaged	60 (35.3)
**Frequency of engagement in employer's wellness program**^d^	n = 110
Once	7 (6.4)
Daily	17 (15.5)
Weekly	38 (34.5)
Monthly	17 (15.5)
Less than <3 months	14 (12.7)
Quarterly	17 (15.5)
**Weight loss among employees who participated in employer's wellness program for weight loss prior to tirzepatide initiation**^d^	n = 110
Did not lose weight	63 (57.3)
Lost weight	47 (42.7)
Lost weight and maintained weight loss	5 (10.6)
Somewhat lost weight and then regained weight	42 (89.4)
**Weight loss with wellness program**^e^**, pounds**	n = 47
Mean (SD)	14.5 (10.4)
**Weight regained with wellness program**^e^**, pounds**	n = 42
Mean (SD)	19.6 (28.8)

Abbreviations: GLP-1, glucagon-like polypetide-1; N, total number of participants; n, number of participants in the specific category; OM, obesity medication; SD, standard deviation.

Values 1–4 are presented as <5.

Unless otherwise specified, data are presented as n (%).

a Survey question was asked to those who said they do not have an employer insurance but do have other insurance that covers medications to treat obesity/overweight.

b Survey question was asked to those who said that their primary full-time employer does offer wellness programs related to weight loss.

c Survey question was asked to those who were required to enroll.

d Survey question was asked to those who said they were currently or have previously enrolled in their employer's wellness program.

e Among those who had lost weight (either regained or maintained after weight loss).

About 33% (n = 170) of participants reported that their full-time employer offered wellness programs related to obesity management. Participants who were required to enroll in wellness programs to receive OMs (n = 23) were asked about which aspect of the programs they had to participate in to be eligible for OMs. Aspects reported were nutritional guidance, support, or counseling (91.3%), weigh-ins or other specific type of health checks (73.9%), health coaching (65.2%), mental health support (30.4%), telehealth (26.1%), gym memberships (21.7%); <5% were required to participate in a specific fitness program (Table 2).

Among those who had engaged in an employer-provided wellness program (n = 110), 30.6% (n = 52) reported being currently engaged at data capture, 34.1% (n = 58) were previously engaged, and overall 35.3% (n = 60) were never engaged. When asked how frequently participants engaged in these wellness programs (n = 110), 34.5% participated weekly; an equal proportion of individuals (15.5%) participated daily, monthly, or quarterly; 12.7% participated for less than a quarter; and only 6.4% participated one time.

Prior to initiating tirzepatide, 42.7% of participants who were currently (at data capture) or previously engaged in a wellness program reported weight loss. Among those who had lost weight, 10.6% (n = 5) maintained their weight loss, while 89.4% (n = 42) regained weight prior to tirzepatide initiation. Participants who had lost weight (who either maintained or regained weight) reported a mean (SD) weight reduction of 14.5 (10.4) pounds and a weight regain of 19.6 (28.8) pounds (Table 2). When asked to rank the key aspects of the wellness program that supported weight loss, most participants reported nutritional guidance, support, or counseling as the primary aspect, followed by weigh-ins or other types of health checks (Fig. S1).

### 3.4 Employer-provided insurance benefits

**Fig. 1:**
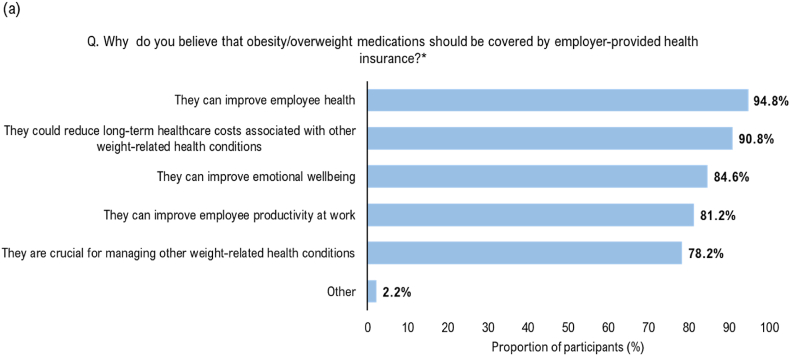

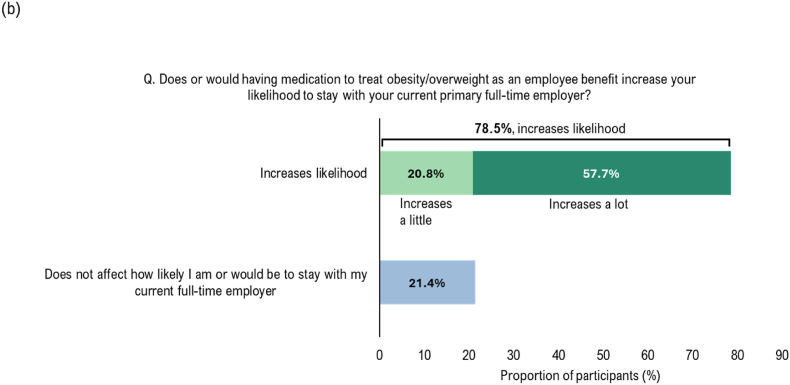

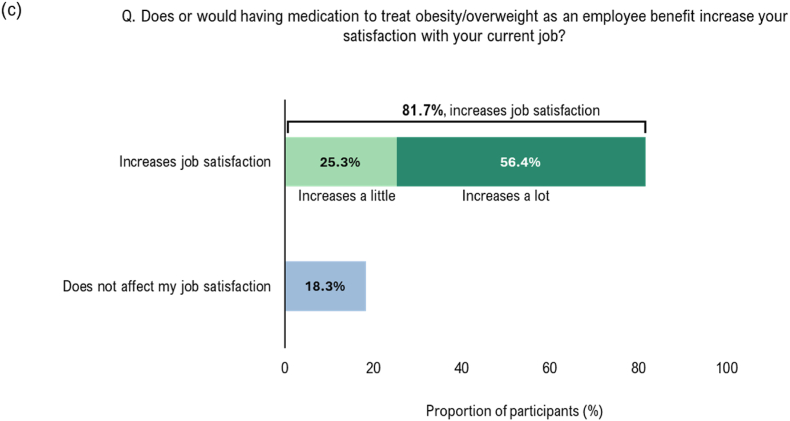

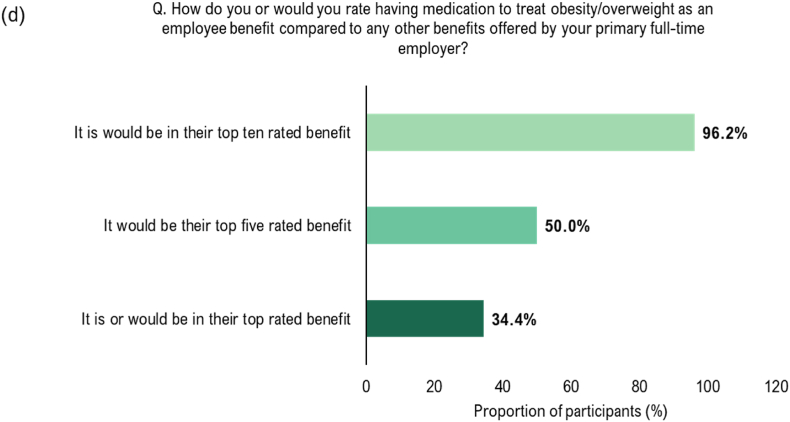

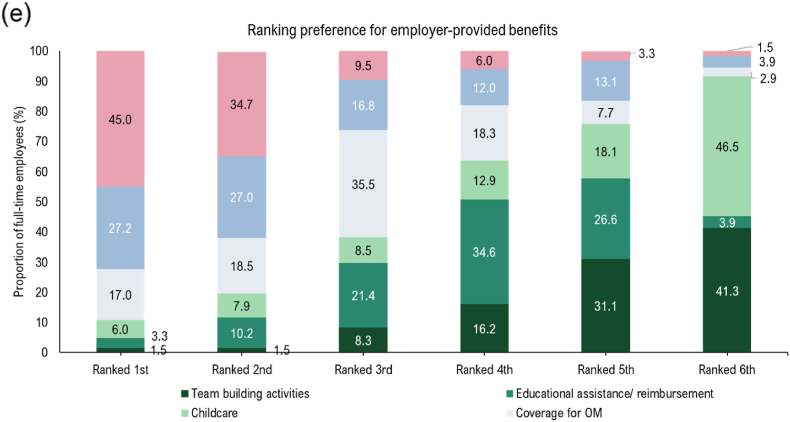
Perceptions regarding employer-provided insurance benefits. *Note: Only asked to those who reported ‘Yes’ to Do you believe that medications to treat obesity/overweight should be covered by employer-provided health insurance (N = 500). Abbreviations: n, number of participants in the specific survey question. 1a: Free text descriptions of “other” were not collected as part of the survey. 1d: 3.9% responded “it is not or would not be my top ten rated benefit”. 1e: Data presented for the survey question: Please rank the following job perks in order of preference list of perks provided as part of employer-provided benefits.

The majority of OM-eligible full-time employees (96.5%) initiating tirzepatide believed that their employer-provided health insurance should include coverage for OMs. Reasons that participants felt that OMs should be covered included the potential to improve employee health, reduce long-term healthcare costs associated with other ORCs, improve emotional wellbeing, increase productivity at work, and that OMs were crucial for managing other ORCs (Fig. 1a). The majority (78.5%) of participants reported that having OMs to treat obesity or overweight as an employee benefit may increase their likelihood of staying with their current employer (Fig. 1b) and 81.7% of participants reported that having OM coverage does or would increase job satisfaction (Fig. 1c). When asked about rating OM coverage as an employee benefit compared to any other employer-provided benefits, 96.2% reported it would be in their top 10 benefits, 50.0% reported it would be in their top 5 benefits, and 34.4% reported it would be their top-rated benefit (Fig. 1d). When assessing preferences for job benefits, the most often top-ranked job benefit was paid time off, ranked first by 45.0% of participants, followed by work-from-home or hybrid work models (27.2%), and coverage for OM (17.0%) (Fig. 1e). Most participants (52.3%) would consider changing their current jobs to get access to OM coverage as an employee benefit.

### 3.5 Work productivity and activity impairment

**Fig. 2:**
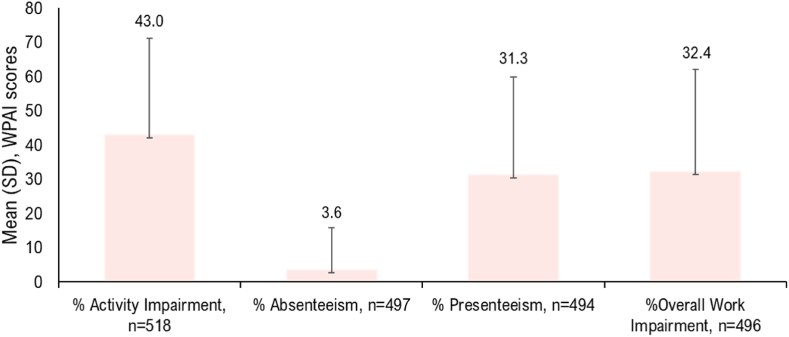
Activity and work impairment among full-time employees. Abbreviations: n, number of participants in the specific category; SD, standard deviation; WPAI, Work Productivity Activity Impairment. WPAI scores represent impairment percentages. Scores range from 0 to 100%; higher percentages indicate greater impairment and less productivity. The overall work impairment score includes time missed (absenteeism) and impairment while working (presenteeism).

On the WPAI questionnaire, participants with obesity reported a mean activity impairment of 43.0% and impairment while working was 31.3% (Fig. 2). Overall work impairment was 32.4%, while the mean time missed from work was 3.6%. In the past 30 days, the mean (SD) of missed days of work was (1.1 [3.1]).

## 4 Discussion

This analysis of full-time employees initiating tirzepatide (Zepbound®) characterizes individuals’ experiences and perceptions regarding employer-sponsored wellness programs, employer-provided benefits, and the impact of OM coverage on retention and satisfaction at work.

Most of the participants reported having employer-provided health insurance at the time of survey completion, and over half had coverage for their OM. The majority believed coverage for OMs should be included as part of their employer-provided health insurance, citing benefits such as improved health, reduced direct and indirect costs, better management of ORCs, and improved work productivity. More than 80% reported that having OM coverage may increase their likelihood of staying with their current employer and increase their job satisfaction. These findings are consistent with another body of evidence indicating that individuals with obesity may have stronger attachment to employers that offer access to essential and desired health benefits. For instance, a recent retrospective claims-based analysis of self-insured employer health plans reported higher retention among employees diagnosed with obesity or ORCs compared with those without [19]. Naber et al. suggested that higher retention may be partly driven by employee's increased reliance on employer-sponsored health benefits when living with obesity or obesity-related complications, as well as other socioeconomic factors [19]. Prior to initiating tirzepatide, 42.7% of individuals who were engaged in wellness programs reported that they lost weight however, 89.4% (n = 42/47) of them regained their lost weight, which underscores the chronic, relapsing nature of obesity and highlights the need for sustained, evidence-based obesity management including ongoing pharmacotherapy as an adjunct to lifestyle intervention to maintain clinically meaningful weight loss.

Beyond perceived value as an employee-benefit by reducing financial and access barriers, employer provided OM coverage may also facilitate treatment initiation, adherence and persistence over time. This is particularly relevant for a chronic disease like obesity, in which sustained long-term treatment is needed to maintain benefits which are not only obesity-related complication outcomes but also functional benefits such as improved wellbeing, daily functioning, and work productivity. The WPAI scores suggest that full-time employees experience the burden of obesity with substantial productivity losses, both at work and in their daily activities. Although, most participants described their jobs as sedentary and absenteeism was low, the data suggest a larger impact on presenteeism. Employees reported that 31.3% of their work performance was impaired, indicating that even when present, their ability to perform tasks effectively was considerably reduced. The overall work impairment (32.4%) and activity impairment (43.0%) highlight the burden on workplace functioning and the impact of obesity, which extends beyond the workplace, affecting routine daily activities. These results are consistent with well-described productivity impacts of chronic diseases such as obesity, suggesting that as obesity and related complications improve with ongoing treatment, work and activity impairment may also improve in parallel [20].

Previously, it has been noted that losing access to OMs creates a sense of anger and helplessness among individuals, impacting their overall wellbeing [21]. Also, consequences of losing health benefits, such as regaining weight with ceasing OMs, may affect individuals’ health and management of ORCs, thereby worsening their conditions, and increasing overall costs [21]. Therefore, it is important to ensure that employees with obesity or overweight continue to have access to OMs through employer-provided benefits that help them sustain their health and maintain long-term weight loss.

### 4.1 Limitations

The self-reported measures and short interval from first dose to survey completion limit causal inference; future longitudinal follow-up should quantify these findings, recognizing that the durability of benefit depends on continued therapy and may be impacted by coverage dynamics. The findings may not be generalizable to the broader population of full-time employees living with obesity in the US. The survey produced answers from a sample of mostly White females, which limits the generalizability of these findings to a broader population; however, this population is similar to what has been reported in other real-world studies [[8], [9], [10], [11]]. Survey data relies on participants’ self-reported information, which may be affected by recall errors, social desirability bias, or misinterpretation of questions. All participants in this survey were receiving tirzepatide at data capture, which could have influenced their views on OM coverage.

## 5 Conclusion

These findings highlight the need for employer and insurer policies that minimize barriers to accessing obesity care and acknowledge the substantial productivity and wellbeing burden experienced by employees with obesity.

• Engagement with employer sponsored wellness programs alone was often insufficient to sustain weight loss prior to tirzepatide initiation, underscoring the need for ongoing, evidence-based pharmacotherapy alongside lifestyle interventions to support long-term obesity management.
• Real-world insights from this study can inform workplace health strategies, benefit design, and counseling approaches that emphasize both clinical and functional value.
• Long-term studies are needed for meaningful improvements in physical, psychosocial, and health-related quality-of-life outcomes and how coverage dynamics shape outcomes, particularly in employed adults living with obesity.

## CRediT author statement

All named authors meet the International Committee of Medical Journal Editors (ICMJE) criteria for authorship for this article, take responsibility for the integrity of the work as a whole, and have given their approval for this version to be published.

THG: Conception of the work, interpretation of data for the work, and critical review of the work for important intellectual content. TAZ: Design of the work, acquisition of data for the work, analysis of data for the work, interpretation of data for the work, drafting, and critical review of the work for important intellectual content. EC: Design of the work, acquisition of data for the work, analysis of data for the work, interpretation of data for the work, drafting, and critical review of the work for important intellectual content. CG: Design of the work, interpretation of data for the work, and critical review of the work for important intellectual content. AV: Design of the work, acquisition of data for the work, interpretation of data for the work, and drafting of the work for important intellectual content. AL, XH, AF, HB: Interpretation of data for the work, drafting, and critical review of the work for important intellectual content. MS: Analysis of data for the work, interpretation of data for the work, and critical review of the work for important intellectual content.

## Data availability statements

The data that support the findings of this study are available from the corresponding author upon reasonable request.

## Compliance with ethics guidelines

All study data were accessed with protocol compliant with the US patient confidentiality requirements, including the Health Insurance Portability and Accountability Act (HIPAA) of 1996 regulations. As all databases used in the study are fully de-identified and compliant with the HIPAA, this study was exempt from Institutional Review Board approval.

Responsibility for editorial decisions and peer review process for this article was delegated to non-author Editors or non-author Associate Editors.

## Declaration of artificial intelligence (AI) and AI-assisted technologies utilized in the writing process

⁠AI tools were used to assist in grammatical assistance and editing during manuscript preparation. No AI tools were used for data analysis, interpretation, or content generation.

## Funding

This study was sponsored by 10.13039/100004312Eli Lilly and Company, Indianapolis, USA.

## Declaration of competing interests

THG, CG, EC, XH, MS are employees and shareholders of Eli Lilly and Company.

TZ, EC, AV are employees and TZ is a stockholder of Clarivate.

HB: research site institution has received research grants from 89Bio, Abbvie, Allergan, Alon Medtech/Epitomee, Aligos, Altimmune, Amgen, Anji Pharma, AstraZeneca, Bioage, Biohaven, Bionime, Boehringer Ingelheim, Carmot, Chorus/Bioage, Corbus, Eli Lilly, Esperion, Evidera, ERX, Fractyl, Gasherbrum, Genentec, GlaxoSmithKline, Graviton, HighTide, Hoffman LaRoche, Home Access, Horizon, Ionis, Kailera, Kallyope, LG-Chem, Marea, Madrigal, Marea, Merck, Metsera, Mineralys, New Amsterdam, Novartis, NovoNordisk, Pfizer, Regeneron, Roche, Satsuma, Selecta, Shionogi, Skye/Birdrock, Terns, TIMI, Veru, Viking, Vivus, Zomagen. Dr. Harold Bays has served as a advisor (e.g., executive/national committee member and/or protocol/drug development advisor) for 89Bio, Altimmune, Amgen, Boehringer Ingelheim, Eva Pharma, Kiniksa, HighTide, Lilly, Nestle, Novo Nordisk, Regeneron, Rivus, Veru, Zomagen, ZyVersa.

AF: Novo Nordisk, Currax, Boehringer Ingelheim, Viking, Zealand, Nestle, Abbott, Rhythm, Eli Lilly and OAC board member.
