# Supplementary material for: Employee perceptions and experiences with tirzepatide treatment for obesity or overweight in the US: Insights from the PERCEPTIONS Survey
Source: Obes Pillars. 2026 Jul 9;19:100298. doi: 10.1016/j.obpill.2026.100298 (PMC13382417; doi:10.1016/j.obpill.2026.100298)
Supplement: Multimedia component 1 [file mmc1.docx]

**Supplementary files**

# **Figure S1. Aspects of employer-provided wellness/weight loss program that supported weight loss among full-time employees**


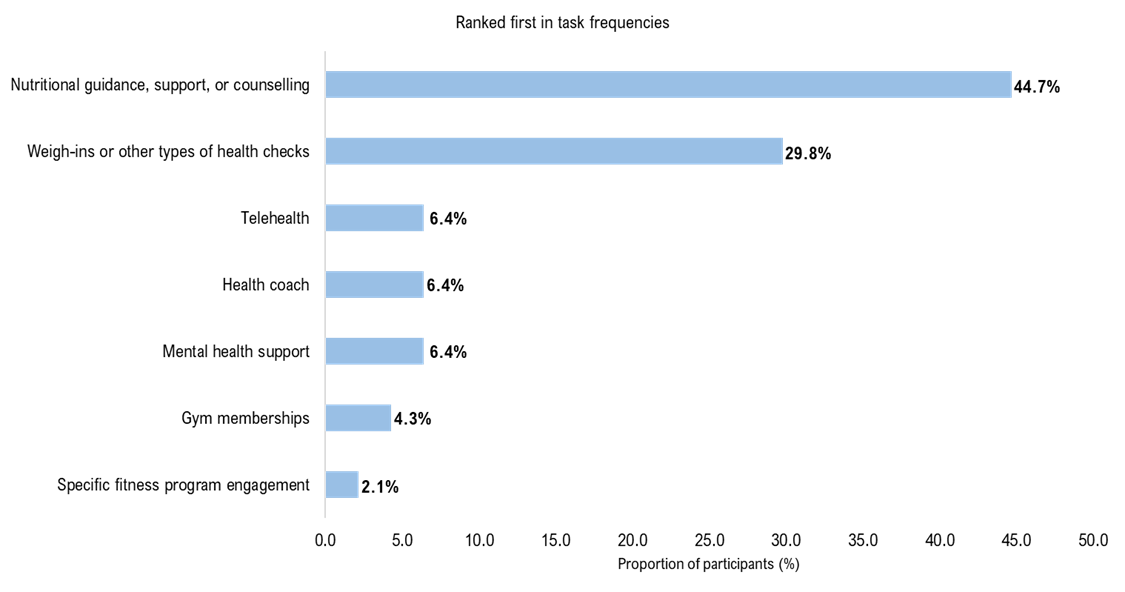


The data presented are in response to the survey question: What aspects of your employer’s weight loss program most supported your weight loss? How would you rank your selected aspects? The question was only asked to those who said they lost weight (either regained or maintained).
